# The Werner syndrome helicase protein is required for cell proliferation, immortalization, and tumorigenesis in Scaffold Attachment Factor B1 deficient mice

**DOI:** 10.18632/aging.100300

**Published:** 2011-03-20

**Authors:** Sophie Lachapelle, Steffi Oesterreich, Michel Lebel

**Affiliations:** ^1^ Centre de Recherche en Cancérologie de l'Université Laval, Hôpital Hôtel-Dieu de Québec, Québec City, Québec, G1R 2J6, Canada; ^2^ Department of Pharmacology and Chemical Biology, University of Pittsburgh Cancer Institute, Magee Women's Research Institute, Pittsburgh, PA 15213, USA

**Keywords:** Werner syndrome, Scaffold Attachment Factor B1, immortalization, senescence

## Abstract

Werner syndrome (WS) is a rare disorder characterized by the premature onset of several pathologies associated with aging. The gene responsible for WS codes for a RecQ-type DNA helicase and is believed to be involved in different aspects of DNA repair, replication, and transcription. We recently identified the Scaffold attachment factor B1 (SAFB1) as a potential interactants in human cells. SAFB1 is a multifunctional protein that binds both nucleic acids and is involved in the attachment of chromatin to the nuclear matrix, transcription, and stress response. Mice lacking SAFB1 exhibit developmental abnormalities in their lungs, high incidence of perinatal lethality, and adults develop different types of tumors. Mouse embryonic fibroblasts from *Safb1*-null animals are immortalized in culture. In this study, mice with a mutation in the helicase domain of the *Wrn* gene were crossed to *Safb1*-null mice. Double homozygous mutant mice exhibited increased apoptosis, a lower cell proliferation rate in their lungs and a higher incidence of perinatal death compared to *Safb1*-null mice. Few double homozygous mutants survived weaning and died before the age of six months. Finally, mouse embryonic fibroblasts lacking a functional Wrn helicase inhibited the immortalization of *Safb1*-null cells. These results indicate that an intact Wrn protein is required for immortalization and tumorigenesis in *Safb1*-null mice.

## INTRODUCTION

Werner syndrome (WS) is a rare autosomal disease characterised by multiple progeroid features like graying and loss of hair, development of diabetes, cataracts, osteoporosis, and cardiovascular disorders at an early age [1]. At the cellular level, WS fibroblasts derived from patients exhibit genomic instability demonstrated by many chromosomal rearrangements, recombination defects, and accumulation of oxidative damage [2-6]. Furthermore, WS fibroblasts reach senescence prematurely in culture compared to age-matched normal fibroblasts [7, 8]. The gene responsible for WS (*WRN*) was identified by positional cloning and the gene product contains a domain homologous to the RecQ-type DNA helicases [9]. The protein also possesses a 3'-5’ exonuclease activity in addition to its 3′-5′ helicase activity [10, 11]. Accumulating evidences indicate that WRN protein is involved in DNA replication/repair, telomere maintenance, and transcription as well [3, 12-17]. Finally, the WRN protein regulates chromatin structures in concert with topoisomerase I to guard against DNA breaks and genomic instability [18].

The Scaffold attachment factor 1 (SAFB1) is also believed to be closely involved in higher order chromatin structure and in the partitioning of chromatin into distinct topologically independent loops [19, 20]. SAFB1 has a RNA binding domain [21], a nuclear localisation domain, Glu/Arg, Ser/Lys, and Gly rich protein interactions regions and a SAF-Box, which is a homeodomain-like DNA-binding motif that interacts with AT-rich scaffold-matrix attachment regions (S/MARs) [22, 23]. S/MARs mediate the attachment of chromatin to the nuclear matrix [23]. SAFB1 is also known to interact with many RNA processing proteins, participating in RNA splicing and the regulation of transcription during cellular stress response [24, 25]. Knockout mice lacking expression of the SABF1 protein exhibit perinatal lethality [26]. More than half of the newborn *Safb1*-null mice display incomplete maturation of the alveoli in their lungs suggesting a dysfunctional oxygen exchange in these animals [26]. The surviving *Safb1*-null mice (approximately 11%) are smaller than heterozygous Safb1^+/−^ mice and are sterile [26]. Interestingly, mouse embryonic fibroblasts (MEFs) derived from Safb1-null embryos show lack of senescence and evidence of cell immortalization in culture [27].

In this study, we examined the impact of a defective Wrn protein (helicase dead Wrn protein) on the phenotype of *Safb1*-null embryos *in vivo* and in cultured cells. We found increased perinatal death in double homozygous mutant mice concomitantly with significant apoptosis in the lungs of such animals. Lastly, we found lack of immortalization of *Safb1*-null MEFs in the absence of a functional Wrn helicase protein *in vitro*.

## RESULTS

### Interaction between WRN and SAFB1 in human cells

**Figure 1.**
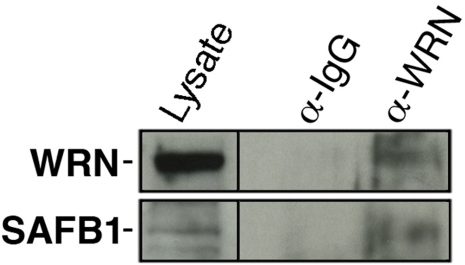
Co-immunoprecipitation of SAFB1 with the WRN protein in HEK 293 cells. Immunoprecipitation was performed with an antibody against the WRN protein. The immuno-precipitate was analyzed by western blot analyses with an antibody against SAFB1. The lysate represents 10% of total proteins in the immunoprecipitation reaction.

Recent mass spectrometry analyses in our laboratory indicated a potential interaction between WRN and SAFB1 proteins [28]. To confirm this result, we immunoprecipitated the WRN protein from HEK 293 cells and analyzed the immunoprecipitate with an antibody against SAFB1. As indicated in Figure 1, SAFB1 protein was co-immunoprecipitated with an antibody against the WRN protein but not with a control IgG. We were unable to immunoprecipitate the SAFB1 protein with the commercial antibody used in this study (data not shown).

### Increased perinatal lethality in *Safb1*-null mice lacking a functional Wrn helicase

To determine the impact of Wrn and Safb1 proteins on the health of mice, mutant animals expressing Wrn protein lacking part of the helicase domain (referred as *Wrn^*Δ*^^hel/^^*Δ*^^hel^* hereafter) were crossed to *Safb1*-null mice. Progenies from the F1 and F2 generations were intercrossed to obtain all potential genotypes. *Safb1*-null mice are known to exhibit perinatal lethality [26]. As indicated in Table 1, crosses between *Safb1^+/−^* heterozygous mice on a *Wrn* wild type background indicated that only 10% of weaned pups were *Safb1^−/−^* homozygous (five males and nine females). This number is close to the 7% of *Safb1^−/−^* homozygous live weaned pups originally described [26]. We also noted a diminution in the number of expected heterozygous animals (*P* < 0.0001). We then calculated the number of *Safb1^−/−^/Wrn^*Δ*^^hel/^^*Δ*^^hel^* double homozygous mutant live pups obtained at weaning. Table 1 indicated that only 3% of weaned pups were *Safb1^−/−^/Wrn^*Δ*^^hel/^^*Δ*^^hel^* double homozygous mutant animals. The number of *Safb1^+/−^/Wrn^*Δ*^^hel/^^*Δ*^^hel^* animals was higher than expected for an unknown reason. We genotyped several dead pups in the cages and found many *Safb1^−/−^/Wrn^*Δ*^^hel/^^*Δ*^^hel^* double homozygous mutants. However, since dead pups were often eaten by the parents, we could not have an exact number for *Safb1^−/−^/Wrn^*Δ*^^hel/^^*Δ*^^hel^* and *Safb1^+/−^/Wrn^*Δ*^^hel/^^*Δ*^^hel^* dead pups. We then crossed *Safb1^+/−^/Wrn^*Δ*^^hel/^^*Δ*^^hel^* females with *Safb1^+/−^/Wrn^*Δ*^^hel/^^*Δ*^^hel^* males and sacrificed pregnant dams at 19 days of gestation. Although we genotyped few *in utero* embryos, the number of *Safb1^−/−^/Wrn^*Δ*^^hel/^^*Δ*^^hel^* animals were close to the expected Mendelian ratio (Table 1). The distribution was not significantly different from the expected Mendelian distribution (*P* = 0.2742). We conclude from these results that most *Safb1^−/−^/Wrn^*Δ*^^hel/^^*Δ*^^hel^* animals died at birth (*P* < 0.0001; significantly different from the expected Mendelian distribution).

**Table 1.** Number and frequencies of wild type and Safb1 mutant pups in litters of *Safb1^+/−^/Wrn^*Δ*^^hel/^^*Δ*^^hel^* and *Safb1^+/−^/Wrn^*Δ*^^hel/^^*Δ*^^hel^* intercrosses

Number of animals	Genotypes +/+ +/− −/−	Ratio +/+ : +/− : −/−	Chi-square test^a^ *P*-value
Wild type*Wrn^+/+^*background			
Adults; 138	47 77 14	1 : 1.6 : 0.3	0.0001479
Mutant*Wrn^*Δ*^^hel/^^*Δ*^^hel^*background			
Adults; 225	62 157 6	1 : 2.8 : 1.6	2.006e-14
Embryos^b^; 97	18 50 29	1 : 2.8 : 1.6	0.2742

a Chi-square test to compare the observed distribution of genotypes with the expected Mendelian 1:2:1 ratio.

b Embryos at 19 days of gestation.

### Life span of *Safb1^−/−^/Wrn^Δhel/Δhel^* double homozygous animals

As indicated in Table 1, only six *Safb1^−/−^/Wrn^*Δ*^^hel/^^*Δ*^^hel^* males (no female) survived weaning (*P* < 0.0001; significantly different from the expected Mendelian distribution). The reason for the absence of females is unknown as the sample size of *Safb1^−/−^/Wrn^*Δ*^^hel/^^*Δ*^^hel^* at weaning was very small (six animals only), but not smaller than *Safb1-*null mice (data not shown). *Safb1-*null animals are smaller than age-matched wild type mice [26]. Two of the *Safb1^−/−^/Wrn^*Δ*^^hel/^^*Δ*^^hel^* males were sacrificed at two months of age to analyze the tissues. Apart from their small size and alopecia (Supplementary Figure 1), no gross abnormality was found in their tissues. The remaining four animals were kept alive in cages to determine their life span. As indicated in Table 2, *Safb1^−/−^/Wrn^*Δ*^^hel/^^*Δ*^^hel^* mice had to be euthanized because they had lost approximately 30% of their weight in one month and were moribund or immobile in the cage. Blood was found in the urine and several of these mice had infection in their eyes. Because of the small sample size, we were unable to determine the exact cause of weight loss in these animals. The oldest *Safb1^−/−^/Wrn^*Δ*^^hel/^^*Δ*^^hel^* mouse had to be sacrificed at 21 weeks of age (ill before six months of age). In contrast, more than 66% of *Safb1*-null/*Wrn^+/+^* animals (six out of nine animals) were still alive by six months of age (data not shown) and more than 95% of *Wrn^*Δ*^^hel/^^*Δ*^^hel^* mice were alive at six months of age [29]. 33% of *Safb1*-null/*Wrn^+/+^* animals had to be euthanized because they had lost more than 25% of their weight in one month and were moribund by six month of age. The phenotype spectrum included myeloid leukemia, blood in urine, infection of eyes, a lung tumor, a tumor in colon, or a tumor in muscle tissue (data not shown). The oldest *Safb1*-null animals died at the age of 21 months (due to an enlarged spleen and a tumor mass in fat tissue).

**Table 2.** Phenotypes of Safb1−/−/Wrn^*Δhel/Δhel*^ mice

Age of diseased animals	Comments
10 weeks	Harderian gland hyperplasia left eye, alopecia
11 weeks	30% loss in weight in one month, infection in one eye, lordokyphosis, moribund, alopecia
15 weeks	25% loss in weight in one month, infection in both eyes
21 weeks	30% loss in weight in one month, blood found in urine

### Increased apoptosis and decreased cell proliferation in the lung tissues of *Safb1^−/−^/Wrn^*Δ*^^hel/^^*Δ*^^hel^* animals

**Figure 2.**
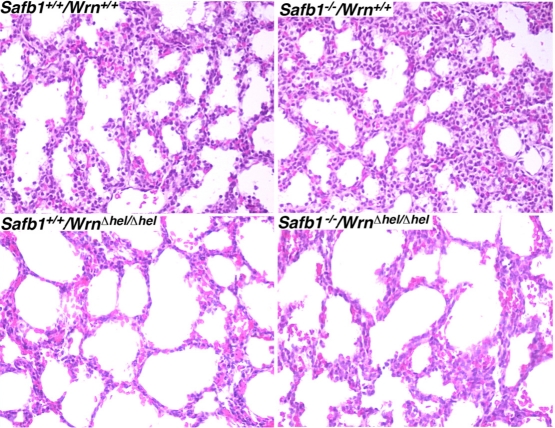
Hematoxilin and eosin staining of lung tissues from 19 days old embryos from the indicated genotypes. Magnification 400X.

**Figure 3.**
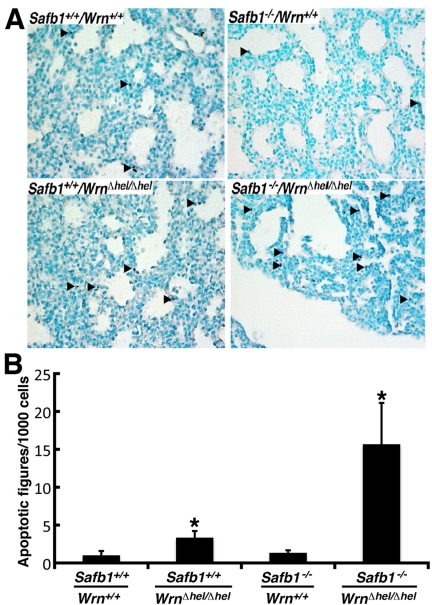
Apoptotic figures in the lung tissues from 19 days old mutant embryos. (**A**) Apoptotic cells detection (TUNEL) assay on 19 days post-coitum embryonic lung sections showing a major increase in the number of apoptotic cells in *Safb1*-null/Wrn^*Δhel/Δhel*^ (or *Safb1^−/−^*/*Wrn*^*Δhel/Δhel*^) embryos compared to the other genotypes. Healthy cells are stained in green (methyl green staining) and apoptotic cells are dark blue. Arrowheads point to representative apoptotic cells. Magnification 400X. (**B**) Average number of apoptotic figures per area of lung sections containing 1000 cells (n=3 embryos for each genotype; *: unpaired student *t*−test *P*-value < 0.05 compared to wild type *Safb1*^*+/+*^/*Wrn*^*+/+*^ animals).

The major phenotype of *Safb1*-null dying pups is the incomplete maturation of the alveoli in the lungs [26]. As indicated in Figure 2, the interalveolar septa of *Safb1*-null pups were larger than those observed in wild type animals. The lack of a functional Wrn helicase caused a decrease in the thickness of the interalveolar septa in *Safb1*-null live pups at birth (compare top and bottom right panels of Figure 2). To get a better quantification of the lung phenotype, we first examined the level of apoptosis in lung tissues using a TUNEL apoptosis kit. TUNEL assays were performed on the lungs of 19 days old embryos. The number of apoptotic cells was estimated on lung sections of three embryos for each genotype (Figure 3A). This number was nearly 16-fold higher in *Safb1-*null/*Wrn^*Δ*^^hel/^^*Δ*^^hel^*lung samples than in wild type and *Safb1-*null samples (unpaired student *t*-test *P*-value < 0.05 compared to wild type *Wrn^+/+^/Safb1^+/+^*embryos) (Figure 3B). The number of apoptotic cells in the lungs of 19 days old *Wrn^*Δ*^^hel/^^*Δ*^^hel^*embryos was approximately three-fold higher than in the lungs of wild type animals (Figure 3B). There was no significant increase in apoptotic figures in the lungs of *Safb1*-null mice compared to wild type animals. These results indicate that there was more cell death in the lungs of *Safb1^−/−^/Wrn^*Δ*^^hel/^^*Δ*^^hel^* embryos at birth than the other genotypes. Cell death was homogeneously distributed across the whole embryonic lung tissue of *Safb1*-null/*Wrn^*Δ*^^hel/^^*Δ*^^hel^*individuals.

**Figure 4.**
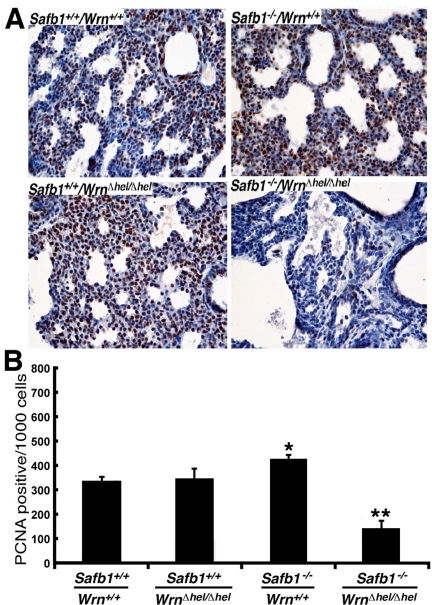
Proliferating cells (stained with an antibody against PCNA) in the lung tissues from 19 days old mutant embryos. (**A**) Example of PCNA stained cells with DAB (brown color) in 19 days post-coitum embryonic lung sections (stained with hematoxilin) showing a major decrease in cell proliferation in *Safb1-*null*/Wrn^Δhel/Δhel^*embryos compared to the other genotypes. Magnification 400X. (**B**) Average number of apoptotic figures per area of lung sections containing 1000 cells (n=3 embryos for each genotype) (*: unpaired student *t*-test *P*-value < 0.001 compared to wild type *Safb1^+/+^/Wrn^+/+^*animals; **: unpaired student *t*-test *P*-value < 0.0001 compared to wild type *Safb1^+/+^/Wrn^+/+^*animals).

Cell proliferation was also examined in the lungs of 19 days old embryos with an antibody against the proliferation cell nuclear antigen (PCNA). As indicate in Figure 4, the number of PCNA positive stained cells in the lungs of *Safb1*-null mice was 27% greater than in the lungs of wild type animals (unpaired student *t*-test *P*-value < 0.001 compared to wild type *Wrn^+/+^/Safb1^+/+^*embryos). In contrast, the number of PCNA positive cells in the lungs of *Safb1^−/−^/Wrn^*Δ*^^hel/^^*Δ*^^hel^* embryos was 42% lower than wild type animals (unpaired student *t*-test *P*-value < 0.0001 compared to wild type *Wrn^+/+^/Safb1^+/+^*embryos). These results indicate that a deficiency of SAFB1 increased pulmonary cell proliferation, but a deficiency in both Safb1 and Wrn functions significantly decreased cell proliferation and increase cell death in the lungs of mutant embryos. We conclude that the Wrn helicase is required for cell survival and proliferation in the lungs of *Safb1*-null animals.

### Loss of Wrn helicase activity in *Safb1*-null mouse embryonic fibroblasts (MEFs) inhibits immorta-lization

The disruption of Safb1 activity in MEFs leads to cell immortalization in culture [27]. We determined the impact of Wrn regarding this process in MEFs established from *Safb1*-null embryos. Previous data have indicated that *Wrn^*Δ*^^hel/^^*Δ*^^hel^* mutant MEFs acquire a slower growth rate than wild type MEFs with the number of passage in culture [30, 31]. MEFs from three to eight embryos of each genotype were established in six-well plates as described previously [32]. MEFs adhering and filling the wells were transferred onto 100-mm petri dishes. Once MEFs reached confluence, cells were trypsinized and transferred to two 100-mm petri dishes with fresh media. This was considered passage number one. Table 3 shows the maximum passage attained by the MEFs of each genotype *in vitro*. Wild type MEFS (from five embryos) were passaged approximately 18 times (approximately 36 population doublings) before they stopped dividing. *Wrn^*Δ*^^hel/^^*Δ*^^hel^* MEFs (from three embryos) were passaged seven to ten times (14-20 population doublings) before entering crisis and stopped dividing in culture. All *Safb1*-null MEFs (three embryos) were passaged more than 40 times (more than 80 population doublings) and were still growing rapidly in culture. MEFs established from *Safb1^−/−^/Wrn^*Δ*^^hel/^^*Δ*^^hel^* double homozygous mutant embryos (eight embryos) stopped dividing after the fifth or seventh passages.

**Table 3.** Maximum number of passage for each mouse embryonic fibroblast genotype *in vitro*

Genotype	Number of passages	Comments
*Safb1^+/+^/Wrn^+/+^*	18	Approximately 36 population doublings
*Safb1^+/+^/Wrn^Δhel/Δhel^*	10	Approximately 20 population doublings
*Safb1^−/−^/Wrn^+/+^*	no maximum	Still growing after 40 passages
*Safb1^−/−^/Wrn^Dhel/Dhel^*	7	Approximately 14 population doublings

**Figure 5.**
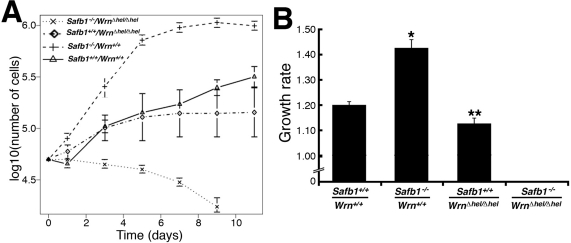
Differential saturation density and growth properties of MEFs. (**A**) Growth curves of MEFs after 7-10 passages in culture (except for *Safb1*-null MEFs, which were measured at passage 24). Cells (5 × 10^4^) from wild type (*Wrn^+/+^/Safb1^+/+^*), *Safb1-*null (*Safb1^−/−^/Wrn^+/+^*), and *Safb1-*null*/Wrn^Δhel/Δhel^* (*Safb1^−/−^/Wrn^Δhel/Δhel^*) embryos were plated in six-well plates as described in materials and methods. Cells were counted by trypan blue exclusion with a hemacytometer. (**B**) Histogram representing the growth rate of MEFs (from at least three embryos for each genotype) calculated from the growth curves in **A**. Bars represent the SEM. (Unpaired student *t*-test: *P < 0.000001 and **P < 0.026577 compared to wild type *Wrn^+/+^/Safb1^+/+^*animals). Growth rates were estimated as described in materials and methods.

Because reduced growth rate is a property associated with *Wrn^*Δ*^^hel/^^*Δ*^^hel^* MEFs [32], we examined this property in fibroblasts derived from *Safb1^−/−^/Wrn^*Δ*^^hel/^^*Δ*^^hel^* double homozygous mutant embryos. We thus measured the average growth rate of MEFs from three embryos of each genotype in culture (after seven to ten passages in culture) (Figure 5). The growth rate of *Safb1*-null MEFs was significantly greater than wild type MEFs (unpaired student *t*-test *P*-value < 0.000001) even after 24 passages in culture (Figure 5B). The calculated doubling time for *Safb1*-null and wild type cells were 34 and 40 hours, respectively. *Safb1*-null MEFs also reached a higher density at confluence than wild type cells (Figure 5A). *Wrn^*Δ*^^hel/^^*Δ*^^hel^* MEFs had a lower growth rate than wild type MEFs (unpaired student *t*-test *P*-value < 0.026). The calculated doubling time for *Wrn^*Δ*^^hel/^^*Δ*^^hel^* MEFs was 43 hours. Finally, *Safb1^−/−^/Wrn^*Δ*^^hel/^^*Δ*^^hel^* double homozygous mutant cells did not grow after seven passages in culture and were detaching from the petri dishes (Figure 5A). All these results indicate that the Wrn helicase is required for the immortalization of *Safb1*-null MEFs in culture.

**Figure 6.**
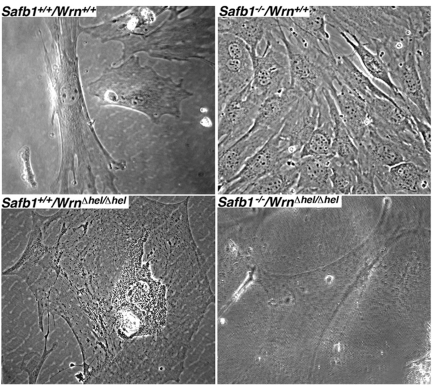
Cellular morphology of MEFs. Representative phase-contrast photographs of wild type, *Wrn^Δhel/Δhel^*, *Safb1-*null, and *Safb1-*null*/Wrn^Δhel/Δhel^* MEFs after the fifth passage in culture. Magnification 600X.

Since the cell density at confluence between *Safb1*-null and wild type MEFs were different, we examined the cellular morphology of each genotype with a phase contrast microscopy (Figure 6). All the MEFs were examined after the fifth passage in culture. *Wrn^*Δ*^^hel/^^*Δ*^^hel^* MEFs were much bigger and flatter than wild type cells (compare top and bottom left panels of Figure 6). *Safb1*-null MEFs were smaller on average than wild type cells (top panels of Figure 6). *Safb1^−/−^/Wrn^*Δ*^^hel/^^*Δ*^^hel^* MEFs were bigger than *Safb1*-null cells but not as big as *Wrn^*Δ*^^hel/^^*Δ*^^hel^* MEFs in culture. These preliminary microscopic observations suggest that cells lacking a functional Wrn helicase had the morphology of senescent cells in culture and this independent of the Safb1 status.

### Lack of Wrn helicase activity in *Safb1*-null MEFs induces senescence

**Figure 7.**
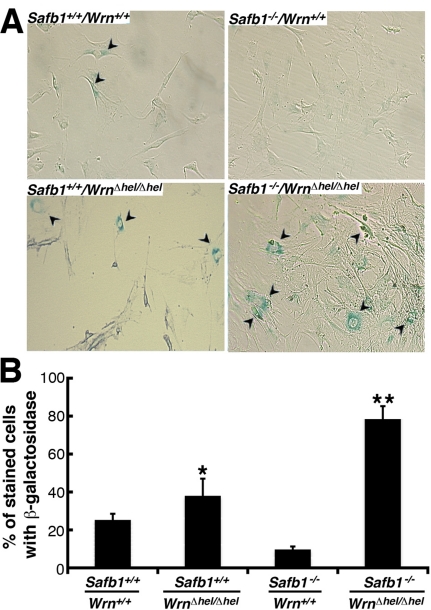
Induction of senescence by loss of Wrn helicase activity in *Safb1-*null MEFs. (**A**) Example of senescence-associated β-galactosidase staining in *Safb1-*null and *Safb1-*null*/Wrn^Δhel/Δhel^* MEFs. Arrowheads point to positive cells. Magnification 100X. (**B**) Percentage of cells stained with senescence-associated β-galactosidase in wild type, *Wrn^Δhel/Δhel^*, *Safb1-*null, and *Safb1-*null*/Wrn^Δhel/Δhel^* MEFs. (Unpaired student *t*-test; *P < 0.045 and ** *P* < 0.022 compared to wild type MEFs). Bars represent SEM.

Replicative senescence of primary fibroblasts *in vitro* consists in the progressive loss of cell division abilities with cellular morphological changes and the accumulation of senescence-associated β-galactosidase activity [33]. We thus compared the number of senescence-associated β-galactosidase positive MEFs of each genotype after the fifth passage in culture. As seen in Figure 7A and B, loss of Wrn helicase activity strongly induced senescence-associated β-galactosidase activity in *Safb1*-null MEFs. The percentage of *Wrn^*Δ*^^hel/^^*Δ*^^hel^* MEFs stained with the senescence-associated β-galactosidase was increased significantly by approximately 1.6-fold compared to wild type cells (unpaired student *t*-test; *P* < 0.022). Finally, the percentage of *Safb1^−/−^/Wrn^*Δ*^^hel/^^*Δ*^^hel^* MEFs stained with the senescence-associated β-galactosidase was increased by three-fold compared to wild type cells (unpaired student *t*-test; *P* < 0.045).

**Figure 8.**
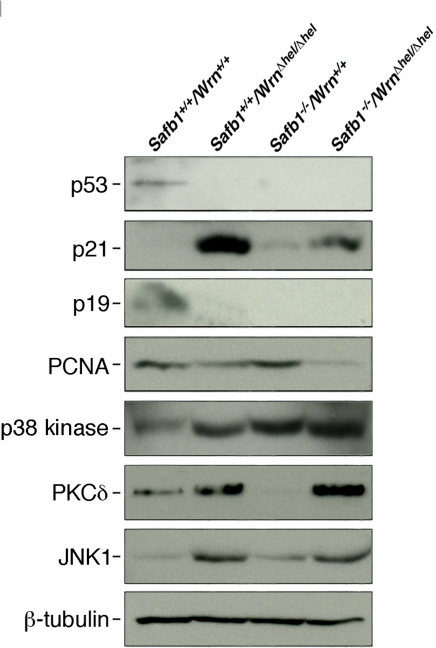
Protein levels of p53, p21Waf1, p19Arf, PCNA, p38 kinase, PKCδ, and JNK1 in MEFs. Whole cell lysates from MEFs of each genotype were analyzed by immunoblotting with antibodies against the indicated proteins. Proteins were extracted from wild type, *Wrn^Δhel/Δhel^*, *Safb1-*null, and *Safb1-*null*/Wrn^Δhel/Δhel^* MEFs. β-tubulin was used as a loading control.

The disruption of Safb1 activity in mouse embryonic fibroblasts leads to the lack of p19Arf induction in culture [27]. The p19Arf protein is a known inducer of senescence in MEFs [34]. We thus examined the levels of several protein markers of senescence, namely p53, p21(Waf1), and p19Arf, as well a marker of cell proliferation (PCNA) in MEFs of each genotype (after at least the sixth passage in culture). The Western blots in Figure 8 show representative results. The p53 protein could be detected in wild type MEFs after a long exposure of Western blot, but we could not detect this protein in any mutant MEFs. The protein p21 was not detected in wild type cells and very weakly expressed in *Safb1*-null MEFs. It could be weakly detected in wild type cells only after a longer exposition of the Western blots (Supplementary Figure S2). It was expressed at high levels in *Wrn^*Δ*^^hel/^^*Δ*^^hel^* MEFs. Although *Safb1-*null*/Wrn^*Δ*^^hel/^^*Δ*^^hel^* double homozygous mutant MEFs entered senescence more rapidly than *Wrn^*Δ*^^hel/^^*Δ*^^hel^* MEFs, p21 expression was greatly reduced compared to *Wrn^*Δ*^^hel/^^*Δ*^^hel^* cells. The p19Arf protein was detected only in wild type MEFs. Finally, PCNA proteins levels were concordant with the growth rates of each cell genotype. Its expression was the highest in *Safb1*-null MEFs and the lowest in *Safb1-*null*/Wrn^*Δ*^^hel/^^*Δ*^^hel^* MEFs (Figure 8). These results indicate that the senescence of *Safb1-*null*/Wrn^*Δ*^^hel/^^*Δ*^^hel^* MEFs is a process independent of p53 and p19Arf.

### Loss of both Wrn helicase and Safb1 activities has an additive effect on the appearance of DNA double strand breaks in MEFs

**Figure 9.**
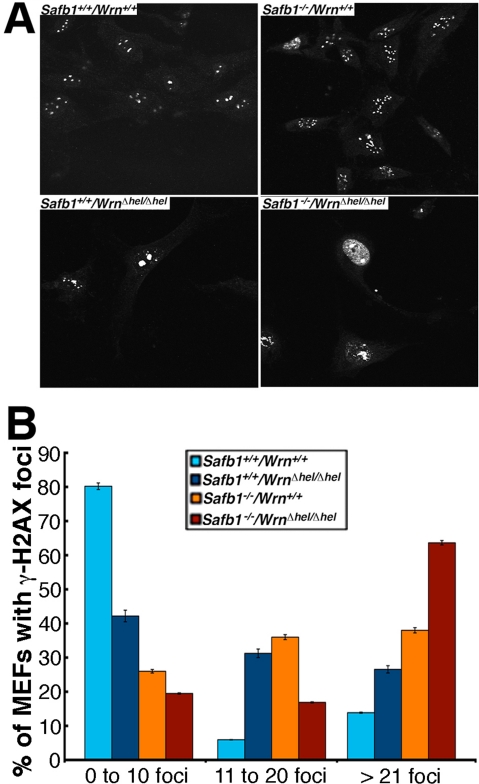
DNA damage in MEFs. (**A**) Examples of nuclear foci detected by immunofluorescence with an antibody against γ-H2AX in MEFs of each genotype Magnification 600X. (**B**) Graph representing the extent of double stranded breaks detected with an antibody against γ-H2AX in MEFs of each genotype. The percentage of cells with more than 0, 10, and 20 γ-H2AX foci were computed from 100 MEFs established from three independent embryos for each genotype (total of 300 cells analyzed/genotype). Bars in the graph represent SEM.

As DNA damage can induce senescence in normal fibroblasts [35], we quantified the levels of DNA strand breaks in the MEFs (three embryos of each genotype) at passage 5 (after approximately 10 population doublings) by immunofluorescence with an antibody against γ-H2AX, which marks double stranded DNA breaks [36]. As indicated in Figures 9A and B, the percentage of *Safb1*-null*/Wrn^*Δ*^^hel/^^*Δ*^^hel^* MEFs with more than 20 γ-H2AX foci reached almost 65% after five passage *in vitro*. Interestingly, even though *Safb1*-null MEFs were immortalizeed, such cells exhibited more γ-H2AX foci than wild type and *Wrn^*Δ*^^hel/^^*Δ*^^hel^* MEFs. More than 25% of *Wrn^*Δ*^^hel/^^*Δ*^^hel^* MEFs showed cells with more than 20 γ-H2AX foci. In contrast, less than 15% of wild type MEFs had nuclei with more than 20 γ-H2AX foci. Approximately 80% of wild type MEFs had less than 10 γ-H2AX foci (Figure 9B). These results indicate that *Safb1*-null*/Wrn^*Δ*^^hel/^^*Δ*^^hel^* MEFs displayed more DNA damage on average than *Safb1*-null, *Wrn^*Δ*^^hel/^^*Δ*^^hel^*, or wild type MEFs.

### The amount of DNA breaks/cell and the number senescent cells correlated with the levels of p38 MAPK and PKCδ respectively, in double homozygous mutant MEFs

**Figure 10.**
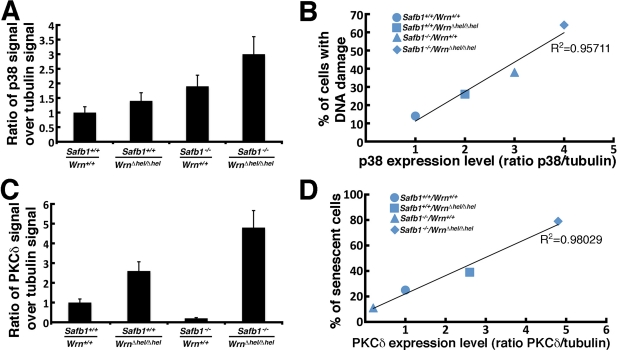
Correlation between p38 and PKCδ kinase levels and DNA damage and senescence in MEFs. (**A**) Scanning analyses of Western blots, expressed as ratio of p38 signal to β-tubulin signal. Bars represent SEM. (**B**) Correlation between the p38 kinase level and DNA damage in the different MEFs. The Pearson's correlation coefficient is indicated. (**C**) Scanning analyses of Western blots, expressed as ratio of PKCδ signal to β-tubulin signal. Bars represent SEM. (**D**) Correlation between the PKCδ level and the percentage of senescent MEFs *in vitro* from the different genotypes. The Pearson's correlation coefficient is indicated.

We finally examined the levels of important kinases involved in cellular stress response and senescence. They include the p38 MAPK [35], the protein kinase C delta (PKCδ), and the Jun Kinase 1 (JNK1) [37, 38]. As shown in Figure 8, the levels of p38 increased in all mutant MEFs compared to wild type cells. High levels were found in *Safb1*-null and *Safb1*-null/*Wrn^*Δ*^^hel/^^*Δ*^^hel^* MEFs with the highest signal in *Safb1*-null/*Wrn^*Δ*^^hel/^^*Δ*^^hel^* MEFs (Figure 10A). An antibody against phosphorylated p38 gave a signal similar to total p38 in MEFs (data not shown). Total p38 levels correlated well with the amount of DNA damage observed in the different genotypic MEFs (Pearson's correlation coefficient R^2^ > 0.95) (Figure 10B).

The PKCδ level in *Wrn^*Δ*^^hel/^^*Δ*^^hel^* MEFs was 2.6-fold higher than wild type cells (Figure 10C). It was 4.8-fold higher in *Safb1*-null/*Wrn^*Δ*^^hel/^^*Δ*^^hel^* MEFs than in wild type cells. In contrast, PKCδ level in *Safb1*-null MEFs were 4.9-fold lower than in wild type cells. Interestingly, the PKCδ protein levels correlated well with the percentage of senescent cells observed in the different genotypic MEFs (Pearson's correlation coefficient R^2^ > 0.98) (Figure 10D).

JNK1 is known to be activated by increased PKCδ levels in cells [38]. We thus examine JNK1 levels in the different MEFs cultures. As indicated in Figure 8, JNK1 was increased by ten-fold in both *Wrn^*Δ*^^hel/^^*Δ*^^hel^* and *Safb1*-null/*Wrn^*Δ*^^hel/^^*Δ*^^hel^* cells. JNK1 level was increased by two-fold in *Safb1*-null MEFs compared to wild type cells. This increase in *Safb1*-null MEFs, however, was not statistically significant (data not shown). JNK1 levels did not correlate with PKCδ levels, the amount of DNA damage, and the percentage of senescent cells in the different MEFs. JNK1 expression was significantly increased only in MEFs lacking Wrn helicase activity.

## DISCUSSION

SAFB1 was originally described as an S/MAR-binding protein important for chromatin organization in the nucleus [22, 23]. It is also involved in transcriptional repression of estrogen receptors [21] and in the formation of perichromatin granules near the nucleoli in response to heat shock [39]. At the physiological level, depletion of Safb1 activity in mice lead to developmental abnormalities in their lungs, high incidence of perinatal lethality, and dwarfism [26]. The WRN protein, in return, is involved in DNA replication/repair, telomere maintenance, and transcription as well [3, 12-17]. The WRN protein also regulates chromatin structures in concert with topoisomerase I to guard against DNA breaks and genomic instability [18]. Mice lacking part of the helicase domain of the murine *Wrn* gene [32] phenocopy the human WS since they exhibit dyslipidemia, type II diabetes, increased systemic reactive oxygen species, increased genomic DNA damage and a 16.5% reduced mean life span compared to wild type animals [29, 40]. Interestingly, recent mass spectrometry analyses in our laboratory indicated a potential interaction between WRN and SAFB1 protein in the context of intact chromatin [28]. Accordingly, we were able to co-immunoprecipitate the SAFB1 protein with antibodies against the WRN protein. We thus decided to cross *Safb1*-null mice with mice lacking part of the helicase domain of the Wrn protein to determine the impact of these proteins on the phenotype of *Safb1*-null*/Wrn^*Δ*^^hel/^^*Δ*^^hel^* mice at the physiological and cellular levels. Since WRN protein will affect the topology of the chromosomes [18] and SAFB1 is involved in chromatin organization [22, 23], we expected to see increased DNA damage in cells lacking the functions of both proteins with deleterious consequences on health span of mice, even though previous spectrometry analyses indicated that both proteins may not interact directly [28]. Indeed, we observed a greater perinatal lethality of the *Safb1*-null*/Wrn^*Δ*^^hel/^^*Δ*^^hel^* pups compared to *Safb1*-null animals (on a normal *Wrn^+/+^* background). We generated 225 live mice at weaning by crossing *Safb1^+/−^/Wrn^*Δ*^^hel/^^*Δ*^^hel^* males and females. Only six males were genotyped double homozygous mutant *Safb1^−/−^/Wrn^*Δ*^^hel/^^*Δ*^^hel^* at weaning. The reason for the absence of females is unknown as the sample size of *Safb1*-null*/Wrn^*Δ*^^hel/^^*Δ*^^hel^* at weaning is very small. We will require to generate an additional 1000 pups from such crosses to get enough live *Safb1*-null*/Wrn^*Δ*^^hel/^^*Δ*^^hel^* mice at weaning and to obtain more significant statistical numbers. Despite the small size sample of *Safb1*-null*/Wrn^*Δ*^^hel/^^*Δ*^^hel^* males, it became obvious that such animals displayed a severe decreased life span. The oldest *Safb1*-null*/Wrn^*Δ*^^hel/^^*Δ*^^hel^* mouse died at the age of 21 weeks. This is in contrast to *Safb1*-null mice that can live up to 84 weeks. *Wrn^*Δ*^^hel/^^*Δ*^^hel^* mice can live up to 100 weeks [29]. All *Safb1*-null*/Wrn^*Δ*^^hel/^^*Δ*^^hel^* males were smaller than wild type animals, had an infection of some sort, and displayed either alopecia or lordokiphosis. More importantly, these mice displayed severe weight loss before becoming completely moribund and immobile, at which point they had to be humanely euthanized. Immunohistochemistry analyses of pulmonary tissues indicated a significant decrease in cell proliferation (measured by counting the number of PCNA positive cells) and a significant increase in apoptotic figures in the same tissue of *Safb1*-null*/Wrn^*Δ*^^hel/^^*Δ*^^hel^* mice compared to all the other genotypes. Thus, the severe reduced life span and some of the premature aging like phenotypes observed in *Safb1*-null*/Wrn^*Δ*^^hel/^^*Δ*^^hel^* mice could be explained by the increased apoptosis and reduced cell proliferation in several tissues like the lungs. Appropriate experiments are warranted to examine global expression profiling and the extent of apoptosis in several tissues of a bigger cohort of *Safb1*-null*/Wrn^*Δ*^^hel/^^*Δ*^^hel^* From the results of this study, we infer that an increased apoptosis together with a reduction in cell proliferation are likely to affect tumor promotion and overall aging in tissues of *Safb1*-null*/Wrn^*Δ*^^hel/^^*Δ*^^hel^* mice.

### An intact Wrn helicase is required for the immortalization of *Safb1*-null MEFs

A major characteristic of *Safb1*-null MEFs is their immortal phenotype *in vitro* [27]. Here, we show for the first time that *Safb1*-null MEFs exhibit an increased number of double stranded breaks *in vitro* compared to wild type MEFs. Such increase in double stranded breaks may lead to mutations inactivating tumor suppressor genes or activating oncogenes. Accordinly, *Safb1*-null mice developed different tumors. Interestingly, the tumor suppressor p53, p19Arf, and p21Waf1 proteins were down regulated or absent in *Safb1*-null MEFs. The decreased expression of these proteins is likely implicated in the immortalized phenotype of *Safb1*-null MEFs. Loss of Wrn activity in *Safb1*-null MEFs, in return, totally inhibited immortalization and induced a senescence phenotype as shown by the increased percentage of *Safb1*-null/*Wrn^*Δ*^^hel/^^*Δ*^^hel^* stained with the senescence-associated β-galactosidase in culture. *Wrn^*Δ*^^hel/^^*Δ*^^hel^* MEFs also showed a lack of p53 and p19Arf expressions. *Wrn^*Δ*^^hel/^^*Δ*^^hel^* MEFs, however, displayed a major increase in p21Waf1 expression unlike *Safb1*-null MEFs. It is important to mention that p21 levels are elevated in prematurely senescent human fibroblasts from WS patients or Ku80-deficient mice aging prematurely [41, 42]. The p21 protein is a potent cyclin-dependent kinase inhibitor that induces senescence of normal and tumor cells *in vitro* in a p53-independent manner [43, 44]. We found that *Safb1*-null/*Wrn^*Δ*^^hel/^^*Δ*^^hel^* MEFs also displayed increased p21 protein expression *in vitro* compared to wild type and *Safb1*-null MEFs. This level, however, was not as high as in *Wrn^*Δ*^^hel/^^*Δ*^^hel^* MEFs. We thus infer from these results that *Safb1*-null/*Wrn^*Δ*^^hel/^^*Δ*^^hel^* cells lacking a functional Wrn helicase activity display a senescence phenotype in a p53- and p19Arf-independent manner and that p21 may play a modest role during this process in Safb1 deficient cells.

We recently reported that a depletion of WRN protein in human cells increase PKCδ activity *in vitro*[45]. In the present study, we found a significant increase in PKCδ protein levels in *Wrn^*Δ*^^hel/^^*Δ*^^hel^* MEFs. This result is consistent with the increased PKCδ protein levels found in liver tissues of *Wrn^*Δ*^^hel/^^*Δ*^^hel^* mice compared to wild type animals [29]. In addition, we found that PKCδ levels were higher in *Safb1*-null/*Wrn^*Δ*^^hel/^^*Δ*^^hel^* cells. In contrast, *Safb1*-null MEFs showed lower levels of PKCδ compared to wild type cells. More importantly, there was a near perfect correlation between PKCδ levels and the percentage of positive β-galactosidase stained cells depending on the genotype of the MEFs under study (Pearson's correlation coefficient R^2^ > 0.98 in Figure 10D). This is a significant finding, as PKCδ plays a key role in the induction of senescence in human breast tumor cells and normal human diploid fibroblasts [37, 38, 46]. The very high levels of PKCδ in *Safb1*-null/*Wrn^*Δ*^^hel/^^*Δ*^^hel^* cells is independent of cellular DNA damage levels as *Safb1*-null MEFs have on average more double strand breaks than wild type and *Wrn^*Δ*^^hel/^^*Δ*^^hel^* MEFs. In contrast, the p38 MAP Kinase correlated very well with the amount of DNA damage within each different MEF genotype (Pearson's correlation coefficient R^2^ > 0.95 in Figure 10B). This is consistent with the observation that p38 is activated in both a p53 dependent and independent manner after DNA damage [47]. Finally, JNK1 level was increased only in MEFs lacking the Wrn helicase activity and this independently of the Safb1 status in cells (Figure 8). These results suggest that the increased expression of PKCδ play a major role in the induction of senescence in MEFs. Additional studies are warranted to determine whether PKCδ inhibits immortalization of *Safb1*-null MEFs and what is the exact molecular mechanism affected in the process.

## METHODS

### Cell line

Human 293 embryonic kidney cells were maintained in DMEM supplemented with 10% fetal bovine serum, penicillin (250 IU/mL), and Streptomycin (250 μl/mL) at 37°C in atmosphere of 5% CO_2_.

### Mice and primary mouse embryonic fibroblasts

Mice lacking part of the helicase domain of the *Wrn* gene were generated by homologous recombination as previously described [32]. In the process, 121 amino acid residues of the Wrn protein were deleted (amino acids 710 to 831; Wrn*^*Δ*^^hel^*). *Wrn^*Δ*^^hel/^^*Δ*^^hel^* homozygous animals were backcrossed onto the pure C57BL/6 genetic background (Harlan Laboratories, Indianapolis, IN) for twelve generations. *Safb1*-null mice were also generated by homologous recombination and had a deletion of exons 7 through 22 [26]. The genetic background of these mice was on a mixed C57BL/6J-129/Sv background. Mice of all possible genotypes were generated by mating homozygous*Wrn^*Δ*^^hel/^^*Δ*^^hel^*individuals with *Safb1*-null mice and inter-crossing the F1 and F2 generations to obtain all four desired genotypes. Genotyping was performed by Southern blotting with appropriate probes [26, 32]. Mice were housed in cages (containing a top filter) on static racks in a conventional animal facility at 22^+^2°C with 40-50% humidity and a 12h light-dark cycle (light cycle: 06:00-18:00h). All mice were fed *ad libitum* with Teklad Global (Madison, WI) 18% protein rodent diet (5% fat). Care of mice was in accordance with the guidelines of the Committee for the protection of animals at the University Laval. Animals were checked every day for any external mass, infection, bleeding, gasping, and overall decrease or change in activity or behavior. Mice that became immobile or moribund were sacrificed for histological examination of their organs as described previously [30].

The generation and maintenance of mouse embryonic fibroblasts have been described previously [30]. Briefly, healthy 14-day old embryos were minced in 6-well plates and maintained in low glucose DMEM supplemented with 10% heat inactivated calf serum at 37°C in an atmosphere of 5% CO_2_. Adherent cells established from embryonic tissues were passaged as soon as they reached confluence. Cell proliferation was determined by plating 5×10^4^ cells in six-well plates. The cultures were maintained for up to 11 days with changing media every other day. Cells were counted on a hemacytometer. The R software version 2.10.1 (http://www.r-project.org/) was used to estimate the growth rate and the associated standard error. Briefly, the logarithm in base 10 was taken from the cell count prior to fitting a linear model of the form log_10_(cell count) = log_10_(50 000) + K*x, where K represents the growth rate and x the day.

### Senescence associated β-galactosidase staining

Senescence-associated β-galactosidase was used as a marker of senescence and cells were stained for this marker as described [48]. The percentage of blue β-galactosidase positive cells was determined by counting at least 200 cells (inverted microscope Nikon TMS).

### Lung histology

Lungs from live 19 days old embryos were fixed in 4% paraformaldehyde and embedded in paraffin. Thin sections were mounted on glass slides and stained with hematoxilin/eosin. TUNEL assays were performed on lung tissue sections for the detection of apoptotic cells using an In situ Apoptosis Detection Kit (R&D Systems, Minneapolis, MN) following the manufacturer's recommendations. Positive cells were counted and photographed. Digital images of tissues were captured using a Leica microscope equipped with a Dage-MTI CCD camera (Mutech Corp., Billerica, MA). To estimate cell proliferation in lung tissues, standard immunohistochemistry with a mouse monoclonal antibody against PCNA (Santa Cruz Biotechnology, Santa Cruz, CA) was performed on the paraffin sections. PCNA positive cells were revealed with diaminobenzidine (DAB).

### Indirect immunofluorescence

Mouse embryonic fibroblasts were grown on glass coverslips for 24 hours, fixed in cold methanol for 10 min, permeabilized with 0.15% Triton X-100 at 4°C for 10 min, washed with PBS, and blocked with 2% milk at 4°C for 30 min. An anti-γ-H2AX monoclonal antibody (Upstate, Lake Placid, NY) diluted in blocking buffer was applied and incubated overnight at 4°C. Coverslips were washed with PBS and incubated with rhodamine-secondary antibody for one hour at room temperature (AmershamPharmacia, Piscataway, NJ). After washing, coverslips were mounted on glass slides and viewed at 60 X magnification on a Nikon inverted diaphot confocal microscope. Images were captured with a BioRad MRC1024 confocal microscopy system and then colored Adobe Photoshop to allow counting the number of foci/cell nucleus.

### Immunoprecipitations and Western blot analysis

Mouse embryonic fibroblasts were lysed in RIPA buffer (50 mM Tris HCl (pH 7.5), 150 mM NaCl, 1% NP-40, 0.1% SDS, 0.5% deoxycholate) for immuno-precipitation and Western blot analyses. A goat polyclonal antibody against WRN (C-19) (Santa Cruz Biotechnology, Santa Cruz, CA) was used for immunoprecipitation and a rabbit polyclonal anti-WRN antibody from US Biologicals (Cleveland, OH) was used for the immunoblots. Rabbit polyclonal antibodies against the phsophorylated and unphosphorylated forms of the p38 MAP kinase were purchased from Cell Signaling Technology (Danvers, MA). A rabbit polyclonal antibody against JNK1 was purchased from AbCam (Cambridge, MA). A mouse monoclonal antibody against SAFB1 was purchased from Upstate Biotechnology (Lake Placid, NY). A horseradish peroxidase conjugated anti-p53 antibody (DO-1), a rat monoclonal antibody against p19(Arf), and a rabbit polyclonal antibody against PKCδ were purchased from Santa Cruz Biotechnology (Santa Cruz, CA). A rabbit polyclonal antibody against p21(Waf1) was purchased from Oncogene Research Products (Boston, MA). Horseradish peroxidase conjugated secondary antibodies and ECL reagents were from Amersham Biosciences (Piscataway, NJ).

## SUPPLEMENTARY FIGURES

Figure S1. A seven weeks old *Safb1^−/−^/WrnΔhel/Δhel* double homozygous mutant mouse compared to an age?matched *Safb1+/−/WrnΔhel/Δhel* littermate (*Safb1* heterozygous mouse on an homozygous *WrnΔhel/Δhel* background). The smallest animal is the *Safb1^−/−^/WrnΔhel/Δhel* mouse in the middle and right panels. Alopecia in a double homozygous mutant mouse is visible on the photograph (left panel).

Figure S2. Protein levels of p21 in MEFs. Longer exposition (3 min with ECL reagents) of the p21 Western blot in figure 8. β-tubulin was used as a loading control.
