# Supplementary figures and images for: The Werner syndrome helicase protein is required for cell proliferation, immortalization, and tumorigenesis in Scaffold Attachment Factor B1 deficient mice
Source: Aging (Albany NY). 2011 Mar 20;3(3):277–90. doi: 10.18632/aging.100300 (PMC3091521; doi:10.18632/aging.100300)

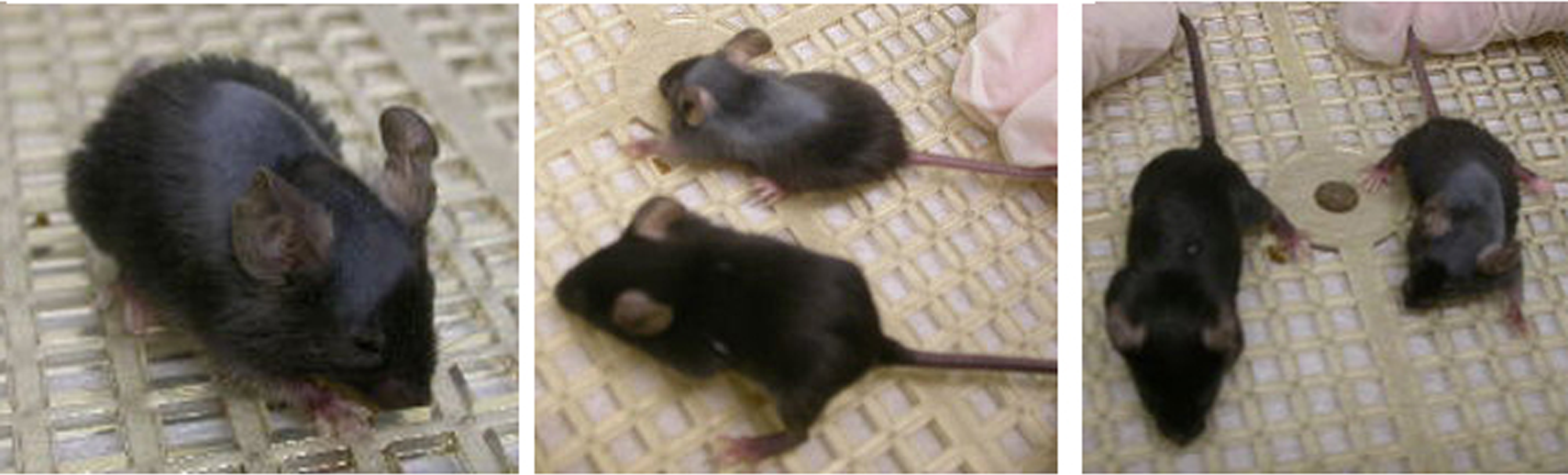

Supplement: Figure S1. — A seven weeks old Safb1−/−/WrnΔhel/Δhel double homozygous mutant mouse compared to an age?matched Safb1+/−/WrnΔhel/Δhel littermate (Safb1 heterozygous mouse on an homozygous WrnΔhel/Δhel background). The smallest animal is the Safb1−/−/WrnΔhel/Δhel mouse in the middle and right panels. Alopecia in a double homozygous mutant mouse is visible on the photograph (left panel). [file aging-03-277-s001.tif]

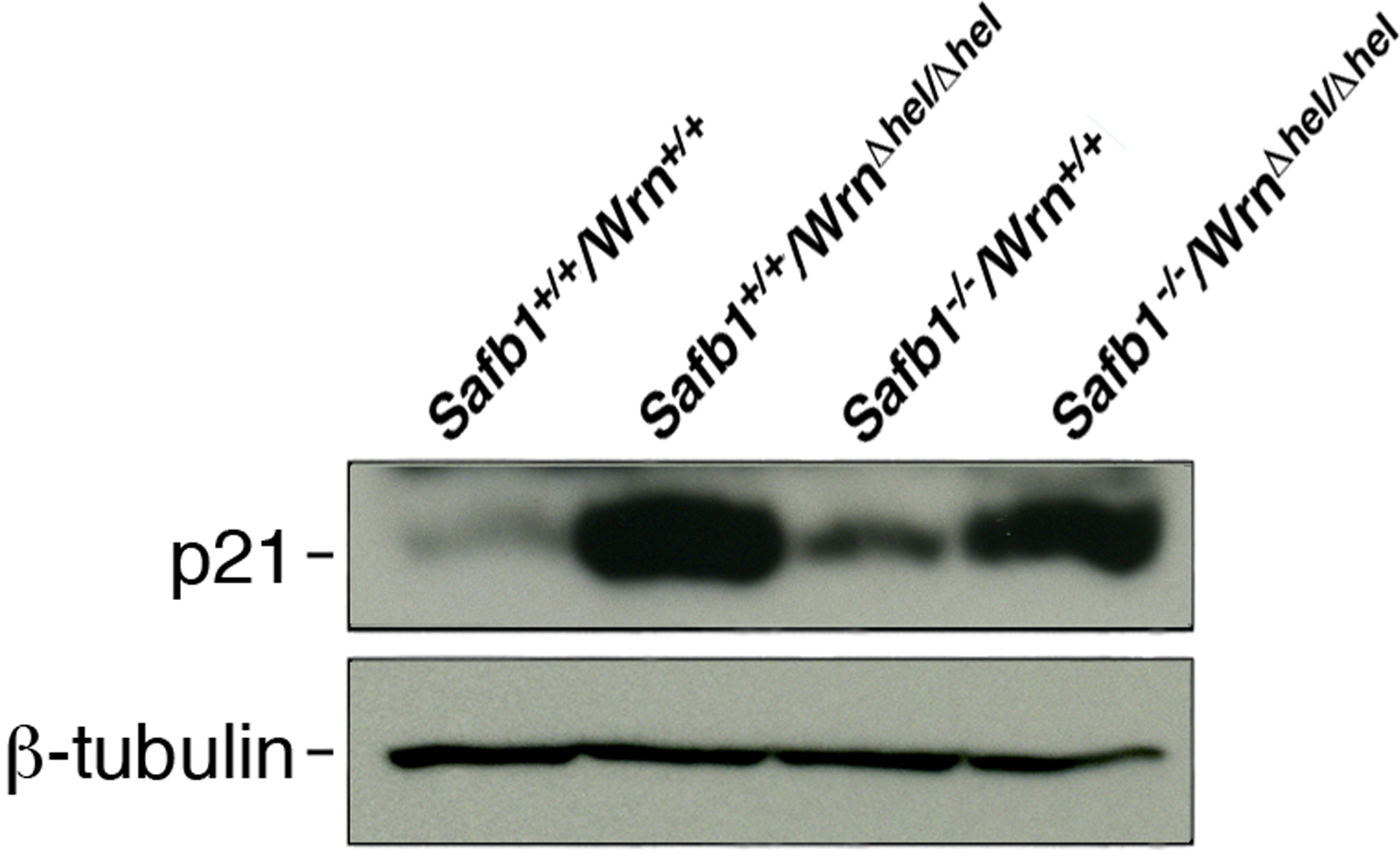

Supplement: Figure S2. — Protein levels of p21 in MEFs. Longer exposition (3 min with ECL reagents) of the p21 Western blot in figure 8. β-tubulin was used as a loading control. [file aging-03-277-s002.tif]
